# LB1581. Exhalation Delivery System with Fluticasone (EDS-FLU) Significantly Reduces Acute Exacerbations and Associated Antibiotic Use in Chronic Rhinosinusitis

**DOI:** 10.1093/ofid/ofac492.1891

**Published:** 2022-12-15

**Authors:** Ramy Mahmoud

**Affiliations:** OptiNose Inc., Yardley, PA

## Abstract

**Background:**

Data suggest chronic rhinosinusitis (CRS) may be the top reason for adult outpatient antibiotic use; of ≈10 million office visits per year for CRS, ≈70% result in antibiotic use. Acute exacerbations of CRS (AECRS) are common, possibly due to persistently impaired mucociliary clearance, and drive use of antibiotics. No drugs have been shown effective for reducing AECRS. ReOpen1 and 2 are randomized controlled trials that evaluated prevention of AECRS with the exhalation delivery system with fluticasone (EDS-FLU; XHANCE®), a novel device delivering topical steroid into chronically inflamed sinonasal regions not typically accessible with standard nasal sprays (eg, past the nasal valve and above the inferior turbinate).

**Methods:**

CRS patients were randomized to EDS-FLU one or two sprays per nostril or placebo twice daily (BID) for 24 weeks. Frequency of AECRS, defined as worsening of at least 1 cardinal symptom of CRS (nasal congestion/obstruction, rhinorrhea, facial pain/pressure, hyposmia/anosmia) for ≥ 3 days requiring escalation of medical care (eg, doctor visit, antibiotic or steroid prescription), was analyzed using pooled data from both trials.

**Results:**

Among 555 patients enrolled, 39.4% were using standard nasal steroids at study entry and 38.8% reported prior sinus surgery. There were 76 AECRS over 24 weeks, almost all (71) resulting in antibiotic use. Patients receiving EDS-FLU had a large reduction in AECRS versus placebo (incidence rate ratio [IRR]=0.39, P=0.001, vs placebo). Reduction was greater at the higher dose (2 sprays/nostril BID) than the lower dose (1 spray/nostril BID): IRR=0.34, P=0.002; IRR=0.44, P=0.012, respectively. 9.9% of low-dose patients and 7.8% of high-dose patients had ≥ 1 AECRS (20 and 15 events, respectively) vs 15.7% receiving placebo (41 events; P=0.012 and P=0.002 vs placebo, respectively). Treatment was well tolerated: adverse events in ≥ 3% of patients and more common in one active group than placebo were epistaxis, COVID-19, headache, and nasopharyngitis.

**Figure:**
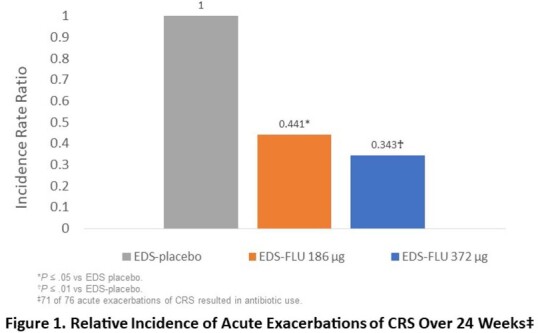

**Figure:**
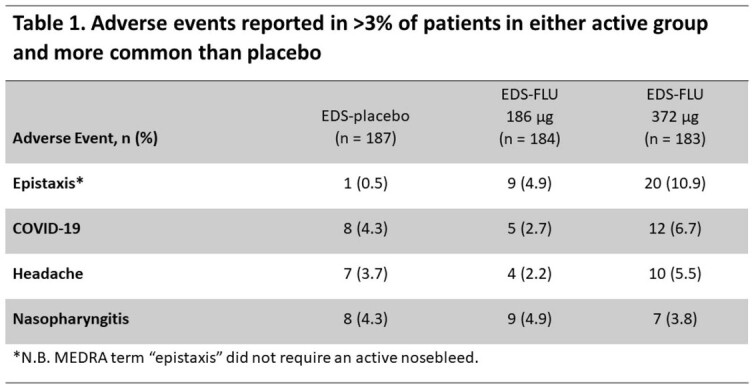

**Conclusion:**

EDS-FLU is the first and only medication shown in randomized controlled trials to significantly reduce acute exacerbations of CRS, offering potential to improve antibiotic stewardship by substantially reducing one of the most common drivers of outpatient antibiotic use.

**Disclosures:**

**Ramy Mahmoud, MD, MPH**, OptiNose Inc.: Employee of OptiNose Inc.

